# 7-Chloro-4-[(*E*)-(3-chloro­benzyl­idene)hydrazinyl]-1λ^4^-quinolinium 3-chloro­benzoate

**DOI:** 10.1107/S1600536809049794

**Published:** 2009-11-25

**Authors:** Marcus V. N. de Souza, R. Alan Howie, Edward R. T. Tiekink, James L. Wardell, Solange M. S. V. Wardell

**Affiliations:** a Instituto de Tecnologia em Farmacos, Fundação Oswaldo Cruz (FIOCRUZ), FarManguinhos, Rua Sizenando Nabuco, 100, Manguinhos, 21041-250 Rio de Janeiro, RJ, Brazil; b Department of Chemistry, University of Aberdeen, Old Aberdeen AB15 5NY, Scotland; c Department of Chemistry, University of Malaya, 50603 Kuala Lumpur, Malaysia; d Centro de Desenvolvimento Tecnológico em Saúde (CDTS), Fundação Oswaldo Cruz (FIOCRUZ), Casa Amarela, Campus de Manguinhos, Av. Brasil 4365, 21040-900 Rio de Janeiro, RJ, Brazil; e CHEMSOL, 1 Harcourt Road, Aberdeen AB15 5NY, Scotland

## Abstract

The title salt, C_16_H_12_Cl_2_N_3_^+^·C_7_H_4_ClO_2_^−^, features a non-planar cation, the dihedral angle between the quinolinium and benzene residues being 18.98 (10)°. The cation adopts an *E* conformation about the C—N bond, and the amine group is oriented towards the quinolinium residue. In the crystal, N—H⋯O hydrogen bonds link two cations with two anions, forming a 20-membered {⋯OCO⋯HNC_3_NH}_2_ synthon. The dimeric units are connected into a linear supra­molecular chain along [100] *via* π–π inter­actions [centroid–centroid distance = 3.5625 (13) Å].

## Related literature

For background information on the pharmacological activity of quinoline derivatives, see: Elslager *et al.* (1969 ▶); Font *et al.* (1997 ▶); Kaminsky & Meltzer (1968 ▶); Musiol *et al.* (2006 ▶); Nakamura *et al.* (1999 ▶); Palmer *et al.* (1993 ▶); Ridley (2002 ▶); Sloboda *et al.* (1991 ▶); Tanenbaum & Tuffanelli (1980 ▶); Warshakoon *et al.* (2006 ▶). For recent studies into quinoline-based anti-malarials, see: Andrade *et al.* (2007 ▶); Cunico *et al.* (2006 ▶); da Silva *et al.* (2003 ▶); de Souza (2005 ▶). For a related crystallographic study on neutral species related to the title compound, see: Kaiser *et al.* (2009 ▶).
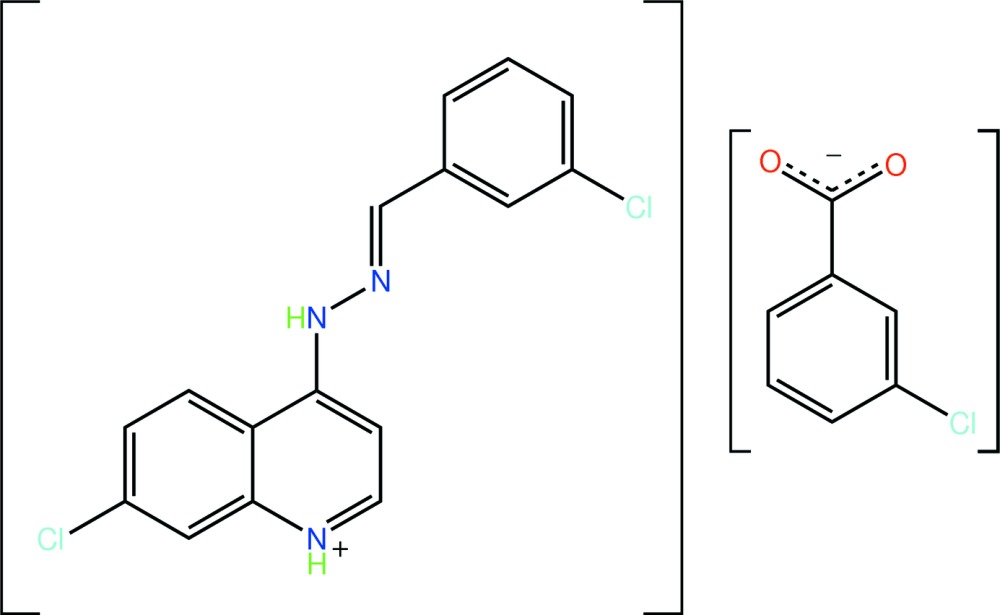


## Experimental

### 

#### Crystal data

C_16_H_12_Cl_2_N_3_^+^·C_7_H_4_ClO_2_^−^
*M* *_r_* = 472.74
Triclinic, 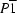

*a* = 8.8777 (2) Å
*b* = 10.7064 (3) Å
*c* = 11.9807 (3) Å
α = 112.5318 (12)°
β = 91.6382 (15)°
γ = 97.4362 (15)°
*V* = 1039.17 (5) Å^3^
*Z* = 2
Mo *K*α radiation
μ = 0.47 mm^−1^
*T* = 120 K
0.06 × 0.04 × 0.03 mm

#### Data collection

Nonius KappaCCD area-detector diffractometer
Absorption correction: multi-scan (*SADABS*; Sheldrick, 2007 ▶) *T*_min_ = 0.922, *T*_max_ = 1.000
16836 measured reflections
4746 independent reflections
3949 reflections with *I* > 2σ(*I*)
*R*_int_ = 0.044

#### Refinement

*R*[*F* ^2^ > 2σ(*F* ^2^)] = 0.047
*wR*(*F* ^2^) = 0.104
*S* = 1.07
4746 reflections
283 parameters
H atoms treated by a mixture of independent and constrained refinement
Δρ_max_ = 0.40 e Å^−3^
Δρ_min_ = −0.45 e Å^−3^

### 

Data collection: *COLLECT* (Hooft, 1998 ▶); cell refinement: *DENZO* (Otwinowski & Minor, 1997 ▶) and *COLLECT*; data reduction: *DENZO* and *COLLECT*; program(s) used to solve structure: *SHELXS97* (Sheldrick, 2008 ▶); program(s) used to refine structure: *SHELXL97* (Sheldrick, 2008 ▶); molecular graphics: *DIAMOND* (Brandenburg, 2006 ▶); software used to prepare material for publication: *publCIF* (Westrip, 2009 ▶).

## Supplementary Material

Crystal structure: contains datablocks global, I. DOI: 10.1107/S1600536809049794/hg2605sup1.cif

Structure factors: contains datablocks I. DOI: 10.1107/S1600536809049794/hg2605Isup2.hkl

Additional supplementary materials:  crystallographic information; 3D view; checkCIF report

## Figures and Tables

**Table 1:** Hydrogen-bond geometry (Å, °)

*D*—H⋯*A*	*D*—H	H⋯*A*	*D*⋯*A*	*D*—H⋯*A*
N1—H1n⋯O2^i^	0.89 (3)	1.76 (3)	2.641 (3)	175 (3)
N2—H2n⋯O1^ii^	0.88	2.00	2.809 (3)	152

Symmetry codes: (i) 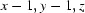; (ii) 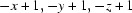.

**Fig. 1.**
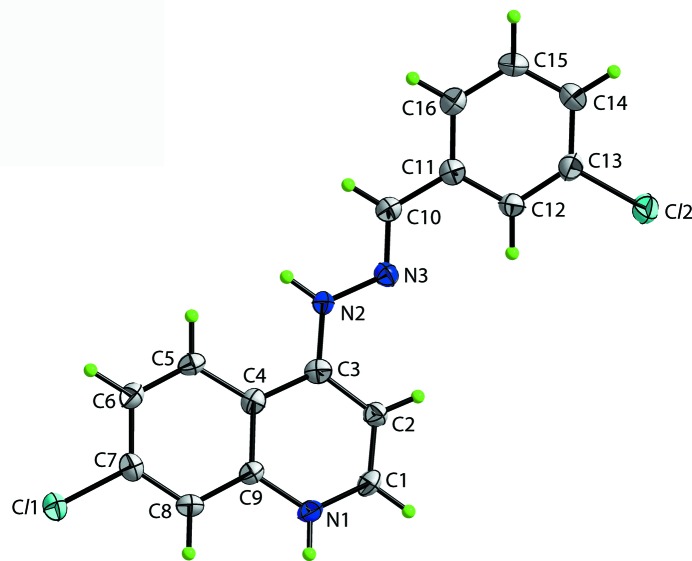
The molecular structure of the cation in (I) showing the atom-labelling scheme and displacement ellipsoids at the 70% probability level.

**Fig. 2.**
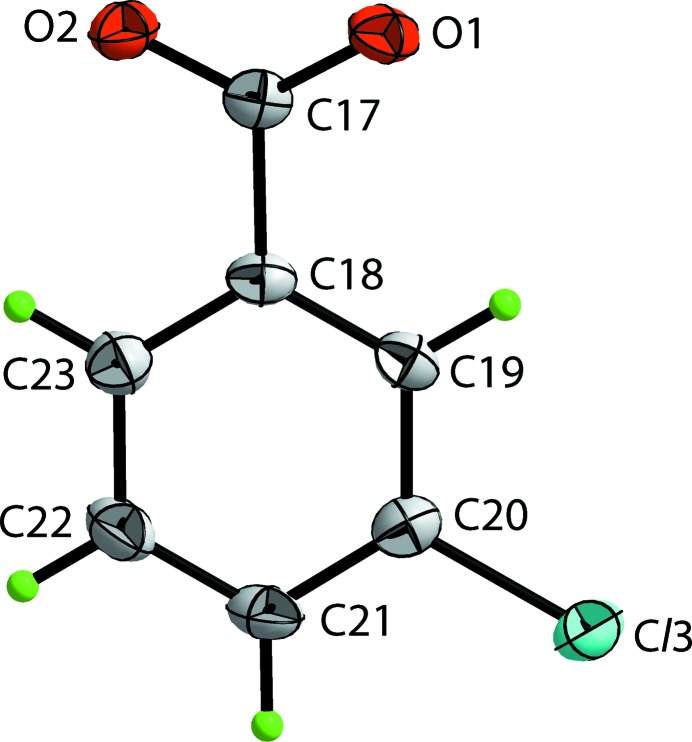
The molecular structure of the anion in (I) showing the atom-labelling scheme and displacement ellipsoids at the 70% probability level.

**Fig. 3.**
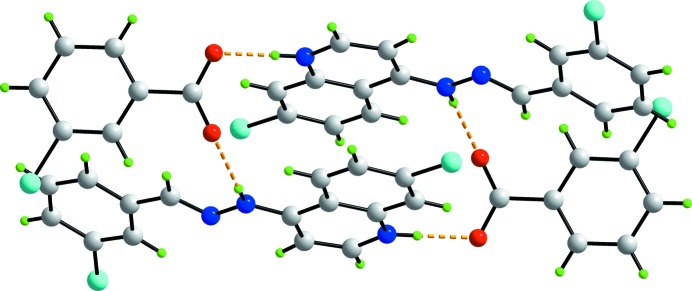
View of the centrosymmetric 20-membered {···OCO···HNC3NH}2 synthon in (I) showing the N–H···O hydrogen bonding as orange dashed lines. Colour code: Cl, cyan; O, red; N, blue; C, grey; and H, green.

**Fig. 4.**
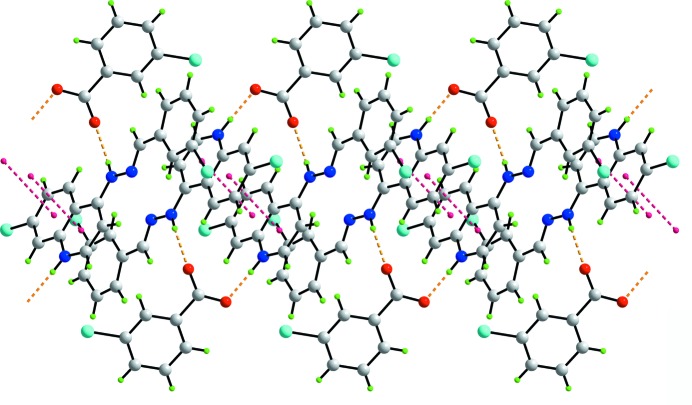
A view of the linear supramolecular chain aligned along [1 0 0] in (I) where the dimeric aggregates illustrated in Fig. 3 are linked by π–π interactions (pink dashed lines).

## supplementary crystallographic information

### Comment

The majority of anti-malarial drugs, such as chloroquine (Tanenbaum & Tuffanelli, 1980), mefloquine (Palmer *et al.*, 1993), primaquine (Elslager *et al.*, 1969) and amodiaquine (Ridley, 2002), possess a quinoline ring, the mainstay of malaria chemotherapy for much of the past 40 years (Font *et al.*, 1997; Kaminsky & Meltzer, 1968; Musiol *et al.*, 2006; Nakamura *et al.*, 1999; Sloboda *et al.*, 1991; Warshakoon *et al.*, 2006). The above motivates our studies aimed towards the development anti-malarial compounds based on the quinoline nucleus (Andrade *et al.*, 2007; Cunico *et al.*, 2006; da Silva *et al.*, 2003; de Souza *et al.*, 2005. The title salt, (I), was prepared as a part of these investigations.

The cation in (I) is twisted about the N2–N3 bond, Fig. 1, as seen in the C3–N2–N3–C10 torsion angle of -168.3 (2) °. This is also reflected in the dihedral angle formed between the quinolinium (maximum deviation = 0.043 (2) for the C2 atom) and benzene planes of 18.98 (10) °. The conformation about the C10═N3 bond is *E*, and the amine-H is oriented towards the quinolinium residue as seen in related structures (Kaiser *et al.*, 2009). The benzoate anion, Fig. 2, is planar with the O1–C17–C18–C19 torsion angle being -10.0 (3) °. The C17–O1, O2 distances in the carboxylate residue are 1.250 (3) and 1.269 (3) Å, respectively, consistent with deprotonation.

The crystal packing is dominated by N–H···O hydrogen bonding, Table 1. A pair of centrosymmetrically related benzoate anions each bridge the quinolinium-H and amine-H atoms of a cation to form a centrosymmetric 20-membered {···OCO···HNC_3_NH}_2_ synthon, Fig. 3. The dimeric units face each other to allow the formation of π–π interactions between the quinolinium residues with the *Cg*(N1, C1—C4, C9)···*Cg*(C4—C9)^i^ distance = 3.5625 (13) Å for i: -*x*, -*y*, 1 - *z*. The net result is the formation of linear supramolecular chains aligned along [1 0 0], Fig. 4.

### Experimental

A solution of 7-chloro-4-hydrazinylquinoline (0.20 g, 1.0 mmol) and 3-chorobenzaldehyde (1.2 mmol) in EtOH (5 ml) was maintained at room temperature overnight and rotary evaporated. The solid residue was washed with cold Et_2_O (3 *x* 10 ml) and recrystallized from EtOH m. pt. 463–465 K, yield 0.24 g The sample for the X-ray study was slowly grown from moist EtOH and the compound isolated was found to be the salt with 3-chlorobenzoic acid. MS/ESI: 315 [C_16_H_10_Cl_2_N_3_], based on ^35^Cl. IR [KBr, cm^-1^] ν 3197 (NH), 1611 and 1552 (CN), 1362 (C—O). The 3-chlorobenzoic acid was subsequently found to be an impurity in the 3-chlorobenzaldehyde reagent.

### Refinement

The quinolinium- and C-bound H atoms were geometrically placed (N–H = 0.88 Å and C–H = 0.95 Å) and refined as riding with U_iso_(H) = 1.2*U*_eq_(C). The amine-bound H atom was located from a difference map and refined (N–H = 0.89 (3) Å) with *U*_iso_(H) = 1.2*U*_eq_(N).

### Crystal data

**Table:**

C_16_H_12_Cl_2_N_3_^+^·C_7_H_4_ClO_2_^−^	*Z* = 2
*M**_r_* = 472.74	*F*(000) = 484
Triclinic, *P*1	*D*_x_ = 1.511 Mg m^−^^3^
Hall symbol: -P 1	Mo *K*α radiation, λ = 0.71073 Å
*a* = 8.8777 (2) Å	Cell parameters from 16230 reflections
*b* = 10.7064 (3) Å	θ = 2.9–27.5°
*c* = 11.9807 (3) Å	µ = 0.47 mm^−^^1^
α = 112.5318 (12)°	*T* = 120 K
β = 91.6382 (15)°	Block, yellow
γ = 97.4362 (15)°	0.06 × 0.04 × 0.03 mm
*V* = 1039.17 (5) Å^3^	

### Data collection

**Table:**

Nonius KappaCCD area-detector diffractometer	4746 independent reflections
Radiation source: Enraf Nonius FR591 rotating anode	3949 reflections with *I* > 2σ(*I*)
10 cm confocal mirrors	*R*_int_ = 0.044
Detector resolution: 9.091 pixels mm^-1^	θ_max_ = 27.5°, θ_min_ = 3.1°
φ and ω scans	*h* = −11→11
Absorption correction: multi-scan (*SADABS*; Sheldrick, 2007)	*k* = −13→13
*T*_min_ = 0.922, *T*_max_ = 1.000	*l* = −15→15
16836 measured reflections	

### Refinement

**Table:**

Refinement on *F*^2^	Primary atom site location: structure-invariant direct methods
Least-squares matrix: full	Secondary atom site location: difference Fourier map
*R*[*F*^2^ > 2σ(*F*^2^)] = 0.047	Hydrogen site location: inferred from neighbouring sites
*wR*(*F*^2^) = 0.104	H atoms treated by a mixture of independent and constrained refinement
*S* = 1.07	*w* = 1/[σ^2^(*F*_o_^2^) + (0.0078*P*)^2^ + 1.9095*P*] where *P* = (*F*_o_^2^ + 2*F*_c_^2^)/3
4746 reflections	(Δ/σ)_max_ = 0.001
283 parameters	Δρ_max_ = 0.40 e Å^−^^3^
0 restraints	Δρ_min_ = −0.45 e Å^−^^3^

### Special details

**Table:**

Geometry. All s.u.'s (except the s.u. in the dihedral angle between two l.s. planes) are estimated using the full covariance matrix. The cell s.u.'s are taken into account individually in the estimation of s.u.'s in distances, angles and torsion angles; correlations between s.u.'s in cell parameters are only used when they are defined by crystal symmetry. An approximate (isotropic) treatment of cell s.u.'s is used for estimating s.u.'s involving l.s. planes.
Refinement. Refinement of *F*^2^ against ALL reflections. The weighted *R*-factor *wR* and goodness of fit *S* are based on *F*^2^, conventional *R*-factors *R* are based on *F*, with *F* set to zero for negative *F*^2^. The threshold expression of *F*^2^ > 2σ(*F*^2^) is used only for calculating *R*-factors(gt) *etc*. and is not relevant to the choice of reflections for refinement. *R*-factors based on *F*^2^ are statistically about twice as large as those based on *F*, and *R*- factors based on ALL data will be even larger.

### Fractional atomic coordinates and isotropic or equivalent isotropic displacement parameters (Å^2^)

**Table:**

	*x*	*y*	*z*	*U*_iso_*/*U*_eq_	
Cl1	−0.20629 (7)	−0.15682 (6)	0.12224 (5)	0.02306 (14)	
Cl2	0.85051 (7)	0.10932 (6)	1.02577 (5)	0.02465 (15)	
N1	0.0669 (2)	−0.2783 (2)	0.43607 (18)	0.0167 (4)	
H1N	−0.007 (3)	−0.349 (3)	0.417 (2)	0.020*	
N2	0.3644 (2)	0.08652 (19)	0.57378 (17)	0.0161 (4)	
H2N	0.3580	0.1499	0.5448	0.019*	
N3	0.4690 (2)	0.1101 (2)	0.67007 (17)	0.0165 (4)	
C1	0.1764 (3)	−0.2566 (2)	0.5229 (2)	0.0176 (5)	
H1	0.1815	−0.3246	0.5550	0.021*	
C2	0.2826 (3)	−0.1393 (2)	0.5677 (2)	0.0169 (5)	
H2	0.3619	−0.1290	0.6269	0.020*	
C3	0.2724 (2)	−0.0350 (2)	0.5250 (2)	0.0150 (4)	
C4	0.1584 (2)	−0.0590 (2)	0.4281 (2)	0.0152 (4)	
C5	0.1406 (3)	0.0354 (2)	0.3736 (2)	0.0160 (4)	
H5	0.2070	0.1203	0.4012	0.019*	
C6	0.0291 (3)	0.0063 (2)	0.2819 (2)	0.0178 (5)	
H6	0.0173	0.0709	0.2469	0.021*	
C7	−0.0678 (3)	−0.1204 (2)	0.2400 (2)	0.0174 (5)	
C8	−0.0558 (3)	−0.2149 (2)	0.2900 (2)	0.0163 (4)	
H8	−0.1226	−0.2996	0.2607	0.020*	
C9	0.0570 (2)	−0.1842 (2)	0.3852 (2)	0.0148 (4)	
C10	0.5321 (3)	0.2345 (2)	0.7240 (2)	0.0183 (5)	
H10	0.5072	0.3008	0.6948	0.022*	
C11	0.6420 (3)	0.2766 (2)	0.8300 (2)	0.0190 (5)	
C12	0.6897 (3)	0.1816 (2)	0.8718 (2)	0.0178 (5)	
H12	0.6519	0.0869	0.8313	0.021*	
C13	0.7928 (3)	0.2280 (2)	0.9730 (2)	0.0197 (5)	
C14	0.8501 (3)	0.3651 (3)	1.0350 (2)	0.0262 (6)	
H14	0.9208	0.3945	1.1043	0.031*	
C15	0.8015 (3)	0.4585 (3)	0.9931 (2)	0.0316 (6)	
H15	0.8388	0.5532	1.0346	0.038*	
C16	0.6988 (3)	0.4153 (3)	0.8908 (2)	0.0274 (6)	
H16	0.6673	0.4803	0.8625	0.033*	
Cl3	0.12274 (6)	0.37851 (6)	0.30843 (6)	0.02426 (14)	
O1	0.67939 (19)	0.66372 (16)	0.43847 (15)	0.0212 (4)	
O2	0.84256 (18)	0.51324 (17)	0.36860 (16)	0.0216 (4)	
C17	0.7100 (3)	0.5460 (2)	0.3827 (2)	0.0165 (5)	
C18	0.5784 (3)	0.4312 (2)	0.3217 (2)	0.0164 (4)	
C19	0.4291 (3)	0.4533 (2)	0.3442 (2)	0.0173 (5)	
H19	0.4091	0.5382	0.4019	0.021*	
C20	0.3100 (3)	0.3500 (2)	0.2816 (2)	0.0182 (5)	
C21	0.3357 (3)	0.2254 (2)	0.1967 (2)	0.0218 (5)	
H21	0.2526	0.1564	0.1535	0.026*	
C22	0.4848 (3)	0.2028 (3)	0.1756 (2)	0.0243 (5)	
H22	0.5041	0.1175	0.1183	0.029*	
C23	0.6058 (3)	0.3051 (2)	0.2385 (2)	0.0206 (5)	
H23	0.7077	0.2889	0.2245	0.025*	

### Atomic displacement parameters (Å^2^)

**Table:**

	*U*^11^	*U*^22^	*U*^33^	*U*^12^	*U*^13^	*U*^23^
Cl1	0.0229 (3)	0.0245 (3)	0.0229 (3)	−0.0014 (2)	−0.0066 (2)	0.0127 (2)
Cl2	0.0323 (3)	0.0204 (3)	0.0219 (3)	0.0074 (2)	−0.0037 (2)	0.0082 (2)
N1	0.0171 (10)	0.0126 (9)	0.0215 (10)	0.0015 (7)	0.0001 (8)	0.0081 (8)
N2	0.0163 (9)	0.0142 (9)	0.0183 (10)	0.0005 (7)	−0.0038 (7)	0.0080 (8)
N3	0.0153 (9)	0.0180 (10)	0.0158 (9)	0.0014 (7)	−0.0011 (7)	0.0067 (8)
C1	0.0199 (11)	0.0147 (11)	0.0211 (12)	0.0062 (9)	0.0018 (9)	0.0092 (9)
C2	0.0163 (11)	0.0156 (11)	0.0181 (11)	0.0020 (9)	−0.0011 (9)	0.0061 (9)
C3	0.0127 (10)	0.0143 (11)	0.0177 (11)	0.0041 (8)	0.0035 (8)	0.0051 (9)
C4	0.0141 (10)	0.0159 (11)	0.0165 (11)	0.0047 (8)	0.0028 (8)	0.0065 (9)
C5	0.0173 (11)	0.0125 (10)	0.0178 (11)	0.0025 (8)	0.0033 (9)	0.0052 (9)
C6	0.0210 (12)	0.0159 (11)	0.0188 (11)	0.0043 (9)	0.0030 (9)	0.0087 (9)
C7	0.0162 (11)	0.0200 (12)	0.0156 (11)	0.0033 (9)	0.0003 (9)	0.0067 (9)
C8	0.0161 (11)	0.0132 (10)	0.0177 (11)	−0.0001 (8)	0.0014 (9)	0.0048 (9)
C9	0.0159 (11)	0.0128 (10)	0.0162 (11)	0.0025 (8)	0.0019 (8)	0.0060 (9)
C10	0.0195 (11)	0.0164 (11)	0.0195 (12)	0.0004 (9)	−0.0005 (9)	0.0085 (9)
C11	0.0211 (12)	0.0179 (11)	0.0179 (11)	0.0009 (9)	−0.0007 (9)	0.0075 (9)
C12	0.0183 (11)	0.0150 (11)	0.0177 (11)	−0.0006 (9)	−0.0001 (9)	0.0050 (9)
C13	0.0231 (12)	0.0198 (12)	0.0190 (12)	0.0044 (9)	0.0018 (9)	0.0103 (10)
C14	0.0321 (14)	0.0227 (13)	0.0207 (12)	−0.0044 (11)	−0.0088 (10)	0.0083 (10)
C15	0.0453 (17)	0.0165 (12)	0.0277 (14)	−0.0068 (11)	−0.0131 (12)	0.0077 (11)
C16	0.0376 (15)	0.0193 (12)	0.0257 (13)	−0.0007 (11)	−0.0082 (11)	0.0115 (11)
Cl3	0.0161 (3)	0.0240 (3)	0.0307 (3)	0.0009 (2)	−0.0005 (2)	0.0094 (3)
O1	0.0227 (9)	0.0133 (8)	0.0253 (9)	0.0007 (6)	−0.0033 (7)	0.0061 (7)
O2	0.0170 (8)	0.0168 (8)	0.0296 (10)	0.0006 (6)	−0.0019 (7)	0.0085 (7)
C17	0.0195 (11)	0.0154 (11)	0.0166 (11)	0.0018 (9)	−0.0025 (9)	0.0092 (9)
C18	0.0180 (11)	0.0145 (11)	0.0175 (11)	−0.0012 (8)	−0.0011 (9)	0.0085 (9)
C19	0.0208 (11)	0.0145 (11)	0.0165 (11)	0.0020 (9)	−0.0003 (9)	0.0065 (9)
C20	0.0168 (11)	0.0195 (12)	0.0205 (11)	0.0012 (9)	0.0002 (9)	0.0108 (9)
C21	0.0222 (12)	0.0180 (12)	0.0208 (12)	−0.0049 (9)	−0.0012 (10)	0.0054 (10)
C22	0.0279 (13)	0.0158 (11)	0.0233 (13)	−0.0006 (10)	0.0033 (10)	0.0024 (10)
C23	0.0208 (12)	0.0195 (12)	0.0211 (12)	0.0019 (9)	0.0029 (9)	0.0077 (10)

### Geometric parameters (Å, °)

**Table:**

Cl1—C7	1.735 (2)	C11—C16	1.395 (3)
Cl2—C13	1.744 (2)	C11—C12	1.398 (3)
N1—C1	1.334 (3)	C12—C13	1.383 (3)
N1—C9	1.373 (3)	C12—H12	0.9500
N1—H1N	0.89 (3)	C13—C14	1.384 (3)
N2—C3	1.348 (3)	C14—C15	1.386 (4)
N2—N3	1.383 (3)	C14—H14	0.9500
N2—H2N	0.8800	C15—C16	1.392 (4)
N3—C10	1.277 (3)	C15—H15	0.9500
C1—C2	1.379 (3)	C16—H16	0.9500
C1—H1	0.9500	Cl3—C20	1.745 (2)
C2—C3	1.407 (3)	O1—C17	1.250 (3)
C2—H2	0.9500	O2—C17	1.269 (3)
C3—C4	1.440 (3)	C17—C18	1.517 (3)
C4—C9	1.416 (3)	C18—C19	1.392 (3)
C4—C5	1.420 (3)	C18—C23	1.394 (3)
C5—C6	1.371 (3)	C19—C20	1.385 (3)
C5—H5	0.9500	C19—H19	0.9500
C6—C7	1.408 (3)	C20—C21	1.385 (3)
C6—H6	0.9500	C21—C22	1.389 (4)
C7—C8	1.372 (3)	C21—H21	0.9500
C8—C9	1.405 (3)	C22—C23	1.392 (3)
C8—H8	0.9500	C22—H22	0.9500
C10—C11	1.465 (3)	C23—H23	0.9500
C10—H10	0.9500		
			
C1—N1—C9	120.8 (2)	C16—C11—C10	118.8 (2)
C1—N1—H1N	119.7 (17)	C12—C11—C10	121.6 (2)
C9—N1—H1N	119.0 (17)	C13—C12—C11	118.9 (2)
C3—N2—N3	119.06 (18)	C13—C12—H12	120.6
C3—N2—H2N	120.5	C11—C12—H12	120.6
N3—N2—H2N	120.5	C12—C13—C14	122.4 (2)
C10—N3—N2	114.66 (19)	C12—C13—Cl2	118.72 (18)
N1—C1—C2	122.5 (2)	C14—C13—Cl2	118.83 (19)
N1—C1—H1	118.8	C13—C14—C15	118.2 (2)
C2—C1—H1	118.8	C13—C14—H14	120.9
C1—C2—C3	119.4 (2)	C15—C14—H14	120.9
C1—C2—H2	120.3	C14—C15—C16	120.9 (2)
C3—C2—H2	120.3	C14—C15—H15	119.6
N2—C3—C2	121.7 (2)	C16—C15—H15	119.6
N2—C3—C4	119.7 (2)	C15—C16—C11	120.0 (2)
C2—C3—C4	118.6 (2)	C15—C16—H16	120.0
C9—C4—C5	117.9 (2)	C11—C16—H16	120.0
C9—C4—C3	118.0 (2)	O1—C17—O2	125.9 (2)
C5—C4—C3	124.1 (2)	O1—C17—C18	118.0 (2)
C6—C5—C4	121.1 (2)	O2—C17—C18	116.1 (2)
C6—C5—H5	119.4	C19—C18—C23	119.6 (2)
C4—C5—H5	119.4	C19—C18—C17	120.1 (2)
C5—C6—C7	119.3 (2)	C23—C18—C17	120.1 (2)
C5—C6—H6	120.4	C20—C19—C18	119.3 (2)
C7—C6—H6	120.4	C20—C19—H19	120.4
C8—C7—C6	121.8 (2)	C18—C19—H19	120.4
C8—C7—Cl1	119.40 (18)	C19—C20—C21	121.6 (2)
C6—C7—Cl1	118.75 (17)	C19—C20—Cl3	119.25 (18)
C7—C8—C9	118.8 (2)	C21—C20—Cl3	119.13 (18)
C7—C8—H8	120.6	C20—C21—C22	119.1 (2)
C9—C8—H8	120.6	C20—C21—H21	120.5
N1—C9—C8	118.6 (2)	C22—C21—H21	120.5
N1—C9—C4	120.5 (2)	C21—C22—C23	120.0 (2)
C8—C9—C4	120.9 (2)	C21—C22—H22	120.0
N3—C10—C11	121.2 (2)	C23—C22—H22	120.0
N3—C10—H10	119.4	C22—C23—C18	120.4 (2)
C11—C10—H10	119.4	C22—C23—H23	119.8
C16—C11—C12	119.6 (2)	C18—C23—H23	119.8
			
C3—N2—N3—C10	−168.3 (2)	C11—C12—C13—C14	0.3 (4)
C9—N1—C1—C2	1.3 (3)	C11—C12—C13—Cl2	179.37 (18)
N1—C1—C2—C3	2.9 (3)	C12—C13—C14—C15	0.0 (4)
N3—N2—C3—C2	−1.5 (3)	Cl2—C13—C14—C15	−179.1 (2)
N3—N2—C3—C4	177.33 (19)	C13—C14—C15—C16	−0.5 (4)
C1—C2—C3—N2	173.8 (2)	C14—C15—C16—C11	0.7 (4)
C1—C2—C3—C4	−5.1 (3)	C12—C11—C16—C15	−0.4 (4)
N2—C3—C4—C9	−175.6 (2)	C10—C11—C16—C15	178.9 (2)
C2—C3—C4—C9	3.3 (3)	O1—C17—C18—C19	−10.0 (3)
N2—C3—C4—C5	3.8 (3)	O2—C17—C18—C19	172.1 (2)
C2—C3—C4—C5	−177.3 (2)	O1—C17—C18—C23	166.8 (2)
C9—C4—C5—C6	−0.3 (3)	O2—C17—C18—C23	−11.1 (3)
C3—C4—C5—C6	−179.7 (2)	C23—C18—C19—C20	−1.1 (3)
C4—C5—C6—C7	−0.9 (3)	C17—C18—C19—C20	175.7 (2)
C5—C6—C7—C8	1.2 (3)	C18—C19—C20—C21	−0.3 (3)
C5—C6—C7—Cl1	−178.96 (18)	C18—C19—C20—Cl3	−179.20 (17)
C6—C7—C8—C9	−0.3 (3)	C19—C20—C21—C22	1.2 (4)
Cl1—C7—C8—C9	179.89 (17)	Cl3—C20—C21—C22	−179.88 (19)
C1—N1—C9—C8	176.9 (2)	C20—C21—C22—C23	−0.7 (4)
C1—N1—C9—C4	−3.2 (3)	C21—C22—C23—C18	−0.7 (4)
C7—C8—C9—N1	179.0 (2)	C19—C18—C23—C22	1.7 (4)
C7—C8—C9—C4	−1.0 (3)	C17—C18—C23—C22	−175.2 (2)
C5—C4—C9—N1	−178.7 (2)	C2—C3—N2—N3	−1.5 (3)
C3—C4—C9—N1	0.8 (3)	C4—C3—N2—N3	177.33 (19)
C5—C4—C9—C8	1.3 (3)	N3—C10—C11—C12	4.1 (4)
C3—C4—C9—C8	−179.3 (2)	N3—C10—C11—C16	−175.2 (2)
N2—N3—C10—C11	178.2 (2)	C19—C18—C17—O1	−10.0 (3)
N3—C10—C11—C16	−175.2 (2)	C19—C18—C17—O2	172.1 (2)
N3—C10—C11—C12	4.1 (4)	C23—C18—C17—O1	166.8 (2)
C16—C11—C12—C13	−0.1 (4)	C23—C18—C17—O2	−11.1 (3)
C10—C11—C12—C13	−179.4 (2)		

### Hydrogen-bond geometry (Å, °)

**Table:**

*D*—H···*A*	*D*—H	H···*A*	*D*···*A*	*D*—H···*A*
N1—H1n···O2^i^	0.89 (3)	1.76 (3)	2.641 (3)	175 (3)
N2—H2n···O1^ii^	0.88	2.00	2.809 (3)	152

Symmetry codes: (i) *x*−1, *y*−1, *z*; (ii) −*x*+1, −*y*+1, −*z*+1.
